# Knowledge of Gestational Diabetes Mellitus Among Adult Females in Al Qassim Province, Saudi Arabia: A Cross-Sectional Study

**DOI:** 10.7759/cureus.53166

**Published:** 2024-01-29

**Authors:** Salem D Almatrafi, Chandra Sekhar

**Affiliations:** 1 Family Medicine, Family Medicine Academy, Qassim Health Cluster, Buraydah, SAU

**Keywords:** al qassim, saudi arabia, women, screening, gestational diabetes, knowledge

## Abstract

Background

Gestational diabetes mellitus (GDM) is a carbohydrate intolerance of variable severity with onset or first recognition during pregnancy; it does not include diabetics who become pregnant or women who become lactosuric. Knowledge of this problem among the public will promote its prevention, screening, and treatment strategies. This study aims to assess the knowledge of GDM regarding its risk factors, screening, treatment, and complications among women in the Al Qassim Province, Saudi Arabia.

Method

A cross-sectional study was conducted among 617 females aged 18 years and older through convenience sampling between October 2022 and January 2023. An online questionnaire was distributed using Google Forms (Google LLC, Mountain View, CA, USA) and WhatsApp (Meta, Menlo Park, CA, USA). Data was entered, cleaned, and analyzed using SPSS Statistics version 27 (IBM Corp., Armonk, NY, USA). Informed consent was obtained from every participant, and the participants' information was kept confidential.

Results

In this study, 52.4% of the women (323/617) had moderate knowledge, and 27.6% (170/617) had excellent knowledge about GDM. Only 13.3% (n = 82) knew the optimum time (24 to 28 weeks of gestational age) for gestational diabetes screening in the absence of risk factors. Moreover, 44.6% (n = 275) knew that insulin is one of the treatments for gestational diabetes, while 45.4% (n = 280) knew that gestational diabetes increases a baby’s risk of obesity and type 2 diabetes mellitus (T2DM) later in life. A statistically significant association was found between the development of GDM with multigravida (19.5%), a BMI of >25 (15%), and age 31 to 45 years (17.8%), with corresponding p-values of 0.001, 0.0001, and 0.0001, respectively.

Conclusion

In this study, almost four-fifths of the study population had moderate to excellent knowledge regarding GDM. However, there is a need to enhance knowledge about optimum screening time and insulin use for gestational diabetes treatment. Therefore, encouraging the existing population to learn more about diabetes education programs and health promotional measures should be undertaken periodically. Further studies are required to support this study's findings.

## Introduction

The definition of gestational diabetes is hyperglycemia with any degree of glucose intolerance appearing as a first recognition during pregnancy; it does not include diabetics who become pregnant or women who become lactosuric [1-3]. The etiology of this disease is not well understood, although genetic and environmental factors have been implicated [4]. Maternal obesity correlates positively with the prevalence of the disease, with later age at childbearing, previous history of gestational diabetes mellitus (GDM), family history of type 2 diabetes mellitus (T2DM), and ethnicity being further risk factors [4,5].

Gestational diabetes mellitus is the most common medical complication during pregnancy, with an increasing prevalence even among young women [5]. According to a systemic review conducted by the International Diabetes Federation in 2022, the global prevalence of gestational diabetes mellitus was 14% (95% confidence interval: 13.97%-14.04%), while it is much higher in the Middle East and North Africa at 27.6% (26.9%-28.4%) [6].

In Saudi Arabia, a study conducted in 2021 at the primary care center of the capital city of Riyadh found that the prevalence of gestational diabetes was 32.6% [7]. A more detailed study conducted at primary healthcare centers in 2020 found that on the glucose challenge test, 36.6% of women showed abnormal values and 6.9% exhibited diagnostic values, while the oral glucose tolerance test indicated impairment in 18.7% of patients and a diagnostic finding in 15% [8].

This problem carries ominous risks for the mother and her offspring. Maternal complications include gestational hypertension, preeclampsia, polyhydramnios, cesarean section, shoulder dystocia, recurrent gestational diabetes, and a seven-fold higher risk of T2DM compared with the general population [4]. Offspring complications include macrosomia, preterm birth, birth injury, shoulder dystocia, neonatal hypoglycemia, neonatal unit admission, and respiratory distress [4].

With these common problems and the health implications for the mother and her fetus, knowledge of this condition becomes essential, particularly regarding risk factors, prevention, screening, detection, and management. In terms of the few available studies in Saudi Arabia, their results regarding GDM knowledge are unpromising [9-11]. In 2022, a cross-sectional study conducted in Jeddah city concluded a lack of knowledge regarding gestational diabetes, as 77.8% of women had poor knowledge [9]. Another study conducted in the same year in Al Madinah Province also found that the knowledge among Saudi women was poor, particularly regarding risk factors, diagnosis, and treatment [10]. Another study in Saudi Arabia showed that 35.5% of the population had adequate knowledge of GDM and its implications, while 34.7% did not understand the condition sufficiently, and 29.8% were unaware of the complications [11].

Jolanta Lis-Kuberka et al. concluded that Polish women had a moderate level of knowledge about the maternal risk factors and adverse neonatal outcomes associated with gestational diabetes [12]. Another study from India found that most participants appeared to have inadequate knowledge regarding the management, physical symptoms, and causes of GDM [13]. A study from Norway found that women with a non-native background and limited education had poor knowledge of gestational diabetes compared with native Norwegian speakers in terms of the effects that gestational diabetes has on the baby, breastfeeding, and the risk of developing T2DM later in life [14]. A study conducted in Al-Khobar stated that the knowledge of gestational diabetes among pregnant women was average; however, the depth of this knowledge was poor [15].

Women's health, diabetes care, and patient education are some of the primary goals of the WHO and the Saudi Kingdom’s 2030 vision. At the time of selecting this research idea, there was no single study evaluating women's knowledge regarding gestational diabetes in the region of Al Qassim. The present study aims to discover the level of knowledge of gestational diabetes and its risk factors, diagnosis, and treatment among women in Al Qassim Province, Saudi Arabia.

## Materials and methods

A cross-sectional study was conducted among the target population of women aged 18 years and older residing in the Al Qassim Province. Before the initiation of the study, the research proposal and execution were approved by the Qassim Regional Ethics Committee (approval no. 607-44-7574). No invasive procedures were involved in this study. Informed consent was obtained from the participant at the time of the online distribution of the questionnaire. The participants' privacy and confidentiality were maintained throughout the research project.

The inclusion criteria for participants were as follows: females aged 18 years and older residing in Al Qassim Province. Females below the age of 18 years, living outside the Qassim region, and those with intellectual disabilities were excluded from the study. A validated questionnaire was provided and approved by the authors of a study conducted in Al Madinah Province in 2022 [10]. Additional questions were added to ensure comprehensiveness and improve validity. The new questions regarding complications of gestational diabetes were reviewed by endocrinologists and experts in family medicine. Opinions from experienced research staff and this study's supervisor were considered. After review by the above team, a pilot test was conducted to study the feasibility of the newly added questions.

The questionnaire mainly contained closed-ended questions, such as age in years, weight in kilograms, and height in centimeters, and a few open-ended questions. An online questionnaire using Google Forms (Google LLC, Mountain View, CA, USA) was distributed to the community via a social media application (WhatsApp, Meta, Menlo Park, CA, USA). As this was an online, self-administered questionnaire, informed consent was attached to the first part of the questionnaire. Once consent was received, the questionnaire appeared. Two reminders for each individual were provided at three-day intervals.

The questionnaire consisted of 18 questions with 'yes,' 'no,' and 'do not know' answers. Each accurate response was given a score of 1, with a total possible score of 18. Answers 'no' and 'do not know' were considered inaccurate and given a 0 score, while 'yes' was considered accurate and given a score of 1. Poor knowledge was determined by a score of 0-6, moderate knowledge received scores of 7-12, and excellent GDM knowledge received scores of 13-18.

In the study conducted in Al Madinah, the authors reported the prevalence of gestational diabetes as poor knowledge in their study to be 53.45% [10]. The same prevalence, a 95% confidence interval, and an absolute precision (alpha) of 5% were adopted in the present study to calculate the sample size. Based on the above calculation, using the WHO statistical software for sample size estimation, the sample estimate was 383. Before the distribution of an online questionnaire, the assumed response rate was low; hence, the questionnaire was distributed to 900 people. Of these, 617 responded, i.e., more than the estimated sample.

As this study was community-based, the questionnaire was designed and distributed using Google Forms via WhatsApp. The questionnaire was delivered to all eligible Al Qassim women aged 18 years and older based on the convenience sampling method. Data was entered, cleaned, and analyzed using SPSS Statistics version 27 (IBM Corp., Armonk, NY, USA). For the continuous variables, means and SD were calculated. For the categorical variables, the chi-square test was applied. Statistical significance was considered at a p-value ≤ 0.05 with 95% confidence limits.

## Results

The questionnaire was distributed to approximately 900 potential study participants, and 617 responses were received. The response rate was 68.5%. In the current study, approximately 570 (92.4%) had heard about gestational diabetes, 20.1% (124/617) of participants had poor knowledge about gestational diabetes, 52.4% (323/617) had moderate knowledge, and 27.6% (170/617) had excellent knowledge. As Table 1 shows, the study population's mean age was 32.87 years ± 10.65. More than half of the people, 52.7% (n = 325), were in the 18 to 30 age group. Approximately 75.9% (n = 468) completed a college education; 69.5% (n = 429) had a family history of diabetes. Furthermore, 34% (n = 210) had gestational diabetes. The study population's mean BMI was 26.38 ± 5.35.

**Table 1:** Demographic and personal characteristics among the adult women in the study population Data presented in the form of n (%) F/H: Family history, GDM: Gestational diabetes mellitus

Variables	N (%)
Age in years ± standard deviation	32.87 ± 10.65
Age category: 18 to 30 years	325 (52.7%)
31 to 45 years	197 (31.9%)
46 to 60 years	89 (14.4%)
More than 60 years	6 (1%)
Nationality: Saudi	608 (98.5%)
Non-Saudi	9 (1.5%)
Area of residence: City	576 (93.4%)
Village	41 (6.6%)
Parity: Never	236 (38.2%)
Once	79 (12.8%)
More than once	302 (48.9%)
Education: Illiterate	3 (0.5%)
School education	91 (14.7%)
College education	468 (75.9%)
Postgraduate	55 (8.9%)
Status: Single	201 (32.6%)
Married	380 (61.6%)
Divorced	29 (4.7%)
Widowed	7 (1.1%)
Health specialization: No	474 (76.8%)
Yes	143 (23.2%)
Any individuals in the home working in health departments: No	335 (54.3%)
Yes	282 (45.7%)
Family history (F/H) of diabetes: No	188 (30.5%)
Yes	429 (69.5%)
F/H of GDM: No	407 (66%)
Yes	210 (34%)
Mean BMI	26.38 ± 5.35

Only 25.4% (n = 157) of participants knew that an increase in the number of pregnancies escalates the risk of developing gestational diabetes. Approximately 61.4% (n = 379) knew that having a first-grade relative with gestational diabetes increased their risk of developing gestational diabetes. Participants with good knowledge (82.2%, n = 507) knew that a past history of gestational diabetes increases the risk of developing gestational diabetes again (Table 2).

**Table 2:** Knowledge of participants regarding GDM risk factors Data is presented in the form of n (%) GDM: Gestational diabetes mellitus, F/H: Family history

GDM risk factors	Correct	Incorrect
Increasing number of pregnancies will increase the risk of developing GDM	157 (25.4%)	460 (74.6%)
Being overweight or obese preconception may contribute to GDM	433 (70.2%)	184 (29.8%)
Excessive weight gain during pregnancy may contribute to GDM	499 (80.9)	118 (19.1%)
Women who had GDM have a higher chance of developing it again	507 (82.2%)	110 (17.8%)
A positive F/H of GDM will increase the risk of developing GDM	379 (61.4%)	238 (38.6%)

Of the study participants, only 13.3% (n = 82) knew the optimum time (24 to 28 weeks of gestational age) for gestational diabetes screening in the absence of risk factors. Another 44.6% (n = 275) knew that insulin is one of the treatments for gestational diabetes. However, the participants displayed good knowledge (89.5%, n = 552) regarding dietary modifications and exercise as part of gestational diabetes treatment (Table 3).

**Table 3:** Participants' awareness on GDM screening and treatment Data is presented in the form of n (%) GDM: Gestational diabetes mellitus

GDM screening	Correct	Incorrect
The optimal time to screen for GDM in the absence of risk factors	82 (13.3%)	535 (86.7%)
Do you think the oral glucose tolerance test is a standard screening test for gestational diabetes?	177 (28.7%)	440 (71.3%)
GDM treatment		
Are dietary modifications and exercise part of the GDM treatment plan?	552 (89.5%)	65 (10.5%)
Do you think insulin injections are one of the treatments available for GDM?	275 (44.6%)	342 (55.4%)
Do you think GDM disappears after delivery?	456 (73.9%)	161 (26.1%)

Around 72.3% (n = 446) of participants knew that gestational diabetes increases the risk of abnormally high birth weight as a complication. However, two-thirds of the study participants (67.3%, n = 415) did not know that gestational diabetes increases the risk of neonatal hypoglycemia. Regarding knowledge of maternal complications, 53.8% (n = 332) knew that gestational diabetes increases the risk of gestational hypertension and preeclampsia, and 64.5% (n = 398) responded that gestational diabetes increases the risk of developing maternal T2DM (Table 4).

**Table 4:** Knowledge about GDM complications in the study group Data is presented in the form of n (%) GDM: Gestational diabetes mellitus, T2DM: Type 2 diabetes mellitus

GDM complications	Correct	Incorrect
Paediatric complications		
GDM increases the risk of excessive birth weight	446 (72.3%)	171 (27.7%)
GDM increases the risk of early (preterm) birth	358 (58%)	259 (42%)
GDM increases the risk of respiratory distress syndrome	209 (33.9%)	408 (66.1%)
GDM increases the risk of low blood sugar for a baby at birth (neonatal hypoglycemia)	202 (32.7%)	415 (67.3%)
GDM increases a baby's risk of obesity and T2DM later in life	280 (45.4%)	337 (54.6%)
Maternal complications		
GDM increases the risk of gestational hypertension and preeclampsia	332 (53.8%)	285 (46.2%)
GDM increases the risk of caesarian delivery	369 (59.8%)	248 (40.2%)
GDM increases the risk of developing maternal T2DM	398 (64.5%)	219 (35.5%)

There was no statistically significant association between various levels of gestational diabetes knowledge and age, nationality, residence, or parity. A statistically significant difference between those who completed postgraduation (49.1% had excellent knowledge) and those who completed school education (20.9% had excellent knowledge) (p < 0.001) was observed. Similarly, 55.9% of the participants with health specialization had an excellent knowledge of gestational diabetes, while only 19% of the non-health specialized participants had excellent knowledge about gestational diabetes, a statistically significant difference (p < 0.001) (Table 5).

**Table 5:** Association of demographic factors with GDM knowledge score in the study population Data is presented in the form of n (%); X^2^ = Chi-square test GDM: Gestational diabetes mellitus, F/H: Family history

Variables	GDM score 0–6 (poor)	GDM score 7–12 (moderate)	GDM score 13–18 (excellent)	X^2 ^and p-value
Age group: 18 to30	70 (21.5%)	165 (50.8%)	90 (27.7%)	X^2 ^−1.575, 6df, p = 0.954
31 to 45	36 (18.3%)	105 (53.3%)	56 (28.4%)	
46 to 60	17 (19.1%)	50 (56.2%)	22 (24.7%)	
60+	1 (16.7%)	3 (50.0%)	2 (33.3%)	
Nationality: Saudi	123 (20.2%)	319 (52.5%)	166 (27.3%)	X^2 ^−1.422, 2df, p = 0.491
Non-Saudi	1 (11.1%)	4 (44.4%)	4 (44.4%)	
Area of residence: City	112 (19.4%)	307 (53.3%)	157 (27.3%)	X^2^ −3.603, 2df, p = 0.165
Rural	12 (29.3%)	16 (39%)	13 (31.7%)	
Parity: Nulliparous	53 (22.5%)	112 (47.5%)	71 (30.1%)	X^2^ −6.325, 4df, p = 0.176
Primigravida	10 (12.7%)	49 (62.0%)	20 (25.3%)	
Multiparous	61 (20.2%)	162 (53.6%)	79 (26.2%)	
Education: Illiterate	1 (33.3%)	1 (33.3%)	1 (33.3%)	X^2 ^−31.7, 6df, p = 0.001
School	33 (36.3%)	39 (42.9%)	19 (20.9%)	
College education	86 (18.4%)	259 (55.3%)	123 (26.3%)	
Postgraduate	4 (7.3%)	24 (43.6%)	27 (49.1%)	
Non-health specialized	116 (24.5%)	268 (56.5%)	90 (19 %)	X^2^ −80.797, 2df, p = 0.0001
Health specialized	8 (5.6%)	55 (38.5%)	80 (55.9%)	
No F/H of GDM	116 (21.1%)	288 (52.5%)	145 (26.4%)	X^2^ −5.005, 2df, p = 0.082
F/H of GDM	08 (11.8%)	35 (51.5%)	25 (36.8%)	

Of the self-reported morbidity status, 22.3% had obesity based on BMI calculation, and 7% had diabetes and polycystic ovarian syndrome (Table 6).

**Table 6:** Prevalence of chronic diseases in the study population Data is presented in the form of n(%)

Self-reported chronic disease	Yes	No	Total
Dyslipidemia	29 (4.7%)	588 (95.3%)	617 (100%)
Thyroid disorders	45 (7.3%)	572 (92.7%)	617 (100%)
Osteoarthritis	26 (4.2%)	591 (95.8%)	617 (100%)
Diabetes mellitus	43 (7%)	574 (93%)	617 (100%)
Hypertension	31 (5%)	586 (95%)	617 (100%)
Polycystic ovarian syndrome	43 (7%)	574 (93%)	617 (100%)
Ischemic heart disease	02 (0.3%)	615 (99.7%)	617 (100%)
Chronic kidney disease	04 (0.6%)	613 (99.4%)	617 (100%)
Obesity	138 (22.3%)	479 (77.7%)	617 (100%)

Almost two-thirds of the study participants (64%, n = 395) chose family and friends as a source of gestational diabetes knowledge. However, only 19.3% (n = 119) obtain information from healthcare providers (Table 7).

**Table 7:** Source of knowledge about GDM in the study group Data is presented in the form of n(%) GDM: Gestational diabetes mellitus

Source of knowledge	Yes	No	Total
Past medical history of GDM	68 (11%)	549 (89%)	617 (100%)
Family/friends	395 (64%)	222 (36%)	617 (100%)
Social media	259 (42%)	358 (58%)	617 (100%)
Books/magazines/newspapers	112 (18.2%)	505 (81.8%)	617 (100%)
TV/radio	67 (10.9%)	550 (89.1%)	617 (100%)
Booklets/brochures	106 (17.2%)	511 (82.8%)	617 (100%)
Healthcare provider	119 (19.3%)	498 (80.7%)	617 (100%)

The mean age was 32.05 years ± 10.44 in the group with no development of gestational diabetes and 39.49 years ± 10.02 in the gestational diabetes development group. A statistically significant association was observed between increasing age and the development of gestational diabetes (t = 5.55, p = 0.001). In Table 8, the chi-square test result for the age group showed a significant association with the development of gestational diabetes (p = 0.0001). Also, a statistically significant association was observed with the development of gestational diabetes in the multigravida (19.5%) and BMI >25 categories (15%), with corresponding p-values of 0.001 and 0.0001, respectively.

**Table 8:** Risk factors and development of GDM in the study population Data is presented in the form of n (%) GDM: Gestational diabetes mellitus

Variables	No GDM development	GDM development	Odds ratio and confidence interval	X^2^, p-value
Age group: 18 to 30 years	309 (95.1%)	16 (4.9%)	4.17, 2.242 - 7.766	X^2^ -22.95, p = 0.001
31 to 45 years	162 (82.2%)	35 (17.8%)		
Parity: Primigravida	74 (93.7%)	5 (6.3%)	3.59, 1.391 - 9.285	X^2^ -7.81, p = 0.01
Multigravida	243 (80.5%)	59 (19.5%)		
BMI categories: Normal (<25)	255 (94.1%)	16 (5.9%)	2.81, 1.571 – 5.051	X^2^ -12.90, p = 0.001
Overweight/obese (>25)	294 (85.0%)	52 (15.0%)		

## Discussion

The present study measured the knowledge of gestational diabetes among women in Al Qassim from October 2022 to January 2023. While gestational diabetes has a considerable impact on the health of the mother and child, it also influences morbidity, disability, and mortality. Early detection of gestational diabetes is paramount for managing and preventing serious complications of this disease.

In this study, almost 20% of the participants had poor knowledge of gestational diabetes, whereas 80% had moderate to excellent gestational diabetes knowledge. A few studies in India also stated that awareness and knowledge about gestational diabetes were fair [16,17]. Fair gestational diabetes knowledge was observed in a study conducted in Taif among the adult female population in Saudi Arabia [18]. Another study in Al-Khobar, Saudi Arabia, expressed that gestational diabetes knowledge among women was 77.7% [19]. A similar result (79.7%) was reported in a United Arab Emirates study [20]. Participants have inadequate awareness and knowledge about gestational diabetes. Additional information is required, such as appropriate gestational diabetes screening time, awareness of adverse outcomes of gestational diabetes, and its prevention at the earliest possible time, which would counter gestational diabetes-associated complications and provide better outcomes.

In this study, only 13.3% of women knew that the optimal time for gestational diabetes screening was 24 to 28 weeks of gestation. Based on the guidelines, a slightly higher percentage (39.1%) was observed in the Al-Khobar study [15]. This higher percentage could be attributed to the fact that the latter was conducted among prenatal women visiting primary healthcare centers, who tend to have more knowledge than the average population. This population-based study includes all female age groups from 18 years and above in the community (age range: 18 to 67 years).

Education also plays a role in raising awareness of gestational diabetes risk factors. In the present study, 49.1% of those who completed postgraduation had excellent gestational diabetes knowledge, whereas only 20.9% of participants who completed school education had excellent gestational diabetes knowledge, a statistically significant difference (p < 0.001). A Dhahran study reported that among secondary school graduates, 15.4% had good knowledge and that among participants with advanced levels of education, 10.3% had good gestational diabetes knowledge. The study also stated that a statistically significant association was observed between gestational diabetes knowledge and different levels of education [19]. Some Indian studies expressed that most participants completed secondary education, that awareness and knowledge were inadequate, and that related education programs and awareness campaigns were required [16-18].

In the current study, the majority of participants (64%) received knowledge about gestational diabetes from their family and friends, and only 19.3% received information from healthcare providers. In the Al-Khobar study, 64.8% of participants were aware of gestational diabetes and mentioned that, overall, the primary source of information was friends and relatives [15]. In contrast, the most common source of knowledge about gestational diabetes observed in the Jeddah study was social media (30.5%) [9]. Approximately 14.4% received information from healthcare providers, a lower percentage than reported in another study in Sharjah [21].

This study shows that less than half of the participants (44.6%) knew that insulin is one of the treatments available fore gestational diabetes. A small percentage (22.9%) of participants who knew about insulin injections were reported in a study conducted among the Qassim women population [22]. Another study conducted in Al Madinah revealed that 22.5% of the participants were aware that insulin is a treatment option for gestational diabetes [10]. A Poland study stated that 36.9% of the population knew diet and insulin use could help manage gestational diabetes [12].

The present study shows that only 25% of participants knew that increased pregnancies would intensify the risk of gestational diabetes development. A study conducted in Wuhan, China, stated that women with more than three pregnancies and women older than 30 with two or three pregnancies had an increased risk of developing gestational diabetes [23]. However, no significant association was observed between increased pregnancies and the development of gestational diabetes in a study conducted in Turkey [24]. Generally, multiparous women tend to be of higher age, and indirectly, multiparous women with a gestational diabetes history tend to have poor control of glucose.

This study indicates that only one-third of the study participants (32.7%) knew that gestational diabetes increases the risk of low blood sugar in the newborn. Similar results (31.7%) were observed in a study conducted at a maternal and children's hospital in Najran, which stated that gestational diabetes increases hypoglycemia at birth [25]. A study conducted in Japan about risk factors for neonatal hypoglycemia and gestational diabetes mentioned that 45% of newborns developed neonatal hypoglycemia [26].

The current study observed that 45.4% of the participants mentioned that gestational diabetes increases a child's risk of obesity and T2DM later in life. Similar to these findings, a study conducted in Greece stated that the chance of obesity among children between two and five years old doubles and is independent of multiple confounding factors [27]. Additionally, gestational diabetes increases the risk of the development of T2DM in offspring later in life, with an eight-fold increase in the risk of developing T2DM observed in a study conducted by Damm in Denmark [28].

Nearly 59.8% of women in this study knew that gestational diabetes increases the risk of cesarean delivery. Similar to these findings, a Brazilian study observed that 57.4% of gestational diabetes mothers with caesarian deliveries had higher odds of additional risk factors due to obesity and previous caesarian deliveries [29]. In the present study, the mean BMI was high (26.38 ± 5.35), and many participants (22.3%) had obesity problems. Many studies have revealed that women with a BMI of 30 and above before pregnancy have a three-fold increased risk of gestational diabetes [30]. In the current study, the risk of gestational diabetes development is significantly associated with an age of more than 30 years, multigravida, and a BMI of more than 25. Similar to this result, a study conducted in Norway reported that the risk of developing gestational diabetes was substantially associated with different age groups (p = 0.05) and BMI (p = 0.04) and not significantly associated with parity (p = 0.87) [14].

The limitations of this study include that the questionnaire was online and obtaining contact information was challenging. The principal investigator tried to obtain more participants through frequent reminders to participants through WhatsApp. Additionally, a self-administered questionnaire presents the possibility of misunderstanding certain questions, which could present an inevitable bias.

## Conclusions

This study's findings show that nearly 80% of the participants had moderate to excellent knowledge of gestational diabetes. However, specific knowledge about optimum screening time and insulin use for gestational diabetes was low. Regarding risk factors for gestational diabetes, approximately 25% of the participants knew that multiple pregnancies increased the risk of developing gestational diabetes. Similarly, for the question related to family members with gestational diabetes increasing the risk of developing gestational diabetes, only six out of 10 responded correctly. Regarding knowledge of gestational diabetes complications, less than 50% of participants knew that it increases the risk of low blood sugar for a baby at birth (neonatal hypoglycemia) and increases a baby's risk of obesity and T2DM later in life. Thus, there is an urgent need to implement population-specific education programs and health promotional measures to enhance awareness about gestational diabetes.
